# Activity of Hawthorn Leaf and Bark Extracts in Relation to Biological Membrane

**DOI:** 10.1007/s00232-013-9566-3

**Published:** 2013-06-18

**Authors:** Aleksandra Włoch, Ireneusz Kapusta, Krzysztof Bielecki, Jan Oszmiański, Halina Kleszczyńska

**Affiliations:** 1 Department of Physics and Biophysics, Wrocław University of Environmental and Life Sciences, Norwida 25, 50-375 Wrocław, Poland; 2 Faculty of Biology and Agriculture, University of Rzeszów, Ćwiklińskiej 2, 35-601 Rzeszów, Poland; 3 Department of Plant Nutrition, Wrocław University of Environmental and Life Sciences, Grunwaldzka 53, 50-375 Wrocław, Poland; 4 Department of Fruit, Vegetable and Grain Technology, Wrocław University of Environmental and Life Sciences, Norwida 25, 50-375 Wrocław, Poland

**Keywords:** Hawthorn extract, Erythrocyte, Natural lipid, Antioxidant activity, Lipid organization, Osmotic resistance

## Abstract

The aim of the study was to identify and determine the percent content of polyphenols in extracts from leaves and hawthorn bark, to examine the effect of the extracts on the properties of the biological membrane as well as to determine their antioxidant activity toward membrane lipids. In particular, a biophysical investigation was conducted on the effect of hawthorn extracts on the osmotic resistance and morphology of erythrocyte cells and on the packing of the heads of membrane lipids. Analysis of the polyphenol content of extracts used the HPLC method. Analysis of the polyphenol composition has shown a dominant share of procyanidins and epicatechin in both extracts. The research showed that the polyphenolic compounds contained in hawthorn extracts are incorporated mainly into the hydrophilic part of the erythrocyte membrane, inducing echinocyte shapes. They also diminish the packing order of the lipid polar heads of the membrane, as evidenced by the lowered generalized polarization values of Laurdan. The substances used induced increased osmotic pressure of erythrocytes, making them less sensitive to changes in osmotic pressure. The presence of the extract compounds in the outer hydrophilic part of the erythrocyte membrane, evidenced by examination of the shapes and packing in the hydrophilic part of membrane, indicates that the substances constitute a kind of barrier that protects the erythrocyte membrane against free radicals, while the membrane-bound extracts do not disturb the membrane structure and, thus, do not cause any side effects.

## Introduction

Hawthorn (*Crataegus* spp.) is a plant that occurs on several continents and has long been used in treating many serious diseases. Present-day medicine uses hawthorn extract to treat low blood pressure and cardiovascular conditions such as paroxysmal tachycardia (Chang et al. 2002). According to literature reports, hawthorn extract lowers the level of low-density lipoprotein cholesterol or triglycerides. It functions as a diuretic agent and has an antiatherosclerotic effect (Chang et al. 2005; Eaton and Kinkade 2003; Kirakosyan et al. 2003).

The healing properties of hawthorn seem to be connected with the antioxidant activity of its polyphenolic compounds. Plant polyphenols are scavengers of free radicals, which are the compounds responsible for oxidizing biological structures, with resultant damage and pathological states of an organism. Free radicals arise in living organisms as a result of not only endogenic agents but also exogenic ones, such as electromagnetic radiation, ultrasound, transitional metal ions and xenobiotics (e.g., herbicides, medicines). Organisms are equipped with enzymatic and nonenzymatic protective systems that ensure cell homeostasis, keeping the free radical concentration at a constant level. If the cell’s protective mechanisms are overstretched, the concentration of free radicals becomes excessive and leads to oxidation of cell components, with resultant damage to proteins, lipids and nucleic acids and disturbed cell structure and function. Such damage connected with oxidation disturbs the functioning of the organism. An important and one of the first places of attack by free radicals is the cell membrane. The excess of free radicals can be dealt with by various compounds consumed by the organism which have the ability to scavenge free radicals. Such is the case with natural polyphenols that are present in various parts of the plant: flowers, leaves, bark, fruits and seeds. Among their properties, the most important is their strong antioxidant action, which places them in the group of natural antioxidants (Kirakosyan et al. 2003; Kondo et al. 2002; Cheel et al. 2007; Sokół-Łętowska et al. 2007; Wojdyło et al. 2007; Jiang et al. 2008; Matkowski et al. 2008; Pajk et al. 2008; Pastor-Cavada et al. 2009; Sun et al. 2009; Stangeland et al. 2009; Ziogas et al. 2010). High antioxidant activity is exhibited by hawthorn extracts.

This research was undertaken as part of an effort to find effective plant antioxidants that have no appreciable side effects. Such compounds when consumed as part of the daily human diet protect the organism against the harmful effect of free radicals. In view of their amphiphilic character, it can be expected that the substances will bind to the lipid phase of the membrane, altering its properties to an extent dependent on the number and depth of membrane penetration (Arora et al. 2000; Nakagawa et al. 2000). In this study the effects of the interaction between polyphenols and the lipid phase of the biological membrane were determined by examining the shape and osmotic resistance of erythrocytes and the packing order of the polar heads of membrane lipids of the erythrocyte membrane. The investigations allowed us to locate the polyphenolic compounds in the erythrocyte membrane and to determine the structural changes that occurred in the membrane hydrophilic region.

This investigation was aimed at identification and determination of the percent share of polyphenolic compounds in hawthorn leaf and bark extracts and at determination of the effect of the extracts on the properties of the biological membrane. In particular, studies were conducted on the influence of the extracts on osmotic resistance, morphology of erythrocytes and packing of the polar heads of membrane lipids. This report also presents the antioxidative activity of hawthorn leaf and bark extracts, whose properties are not as well known as hawthorn fruit extracts. The aim of this research was also to determine the composition and antioxidative activity of extracts from hawthorn leaves and bark toward various objects, from simple systems comprising lipids (linoleic acid) through the lipid phase (membrane lipids) and up to a protein-lipid system (erythrocyte membrane). The antioxidative activity of the extracts was estimated compared to that of 6-hydroxy-2,5,7,8-tetramethylchroman-2-carboxylic acid (Trolox^®^).

## Materials and Methods

The subject of the study was plant extracts from the leaves and bark of hawthorn. Hawthorn stolons were cut from the Garden of Medicinal Plants herbarium of the Medical University of Wrocław, Poland. Bark was cut from stolons using a knife, directly frozen in liquid nitrogen and freeze-dried (24 h; Alpha 1-4 LSC; Christ, Osterode am Harz, Germany). Homogeneous powders were obtained by crushing the dried tissues using a closed laboratory mill to avoid hydration. Powders were kept in a freezer (–80 °C) until extract preparation. The extraction procedure of polyphenols was described previously by Gąsiorowski et al. (1997). Polyphenols were isolated from the bark by extraction with water containing 200 ppm SO_2_, the ratio of this solvent to bark being 3:1 (v/v). The extract was adsorbed on AP 400 resin (Purolite, Llantrisant, UK) for further purification. The polyphenols were then eluted out with 80 % ethanol, concentrated and freeze-dried. The content of polyphenols in individual preparations was determined by the liquid chromatography (ultrahigh-performance liquid chromatography with diode-array detector–mass spectrometry [UPLC-DAD-MS]) method.

The choice of erythrocytes was dictated by the fact that the pig red blood cell membrane is known to be closest to the human erythrocyte membrane with respect to its lipid composition. Fresh pig blood was added to a physiological solution with heparin added. Erythrocyte membranes (ghosts) were prepared using the method of Dodge et al. (1963). Natural lipids were extracted from erythrocyte membranes according to the method described by Maddy et al. (1972).

The 3-(4-(6-phenyl)-1,3,5-hexatrienyl) phenylpropionic acid (DPH-PA) and 6-dodecanoyl-2-dimethylaminonaphthalene (Laurdan) fluorescence probes were purchased from Molecular Probes (Eugene, OR). The oxidation inductor 2,2′-azobis(2-amidinopropane)hydrochloride (AAPH) and Trolox were purchased from Sigma-Aldrich (Steinheim, Germany) and the oxidation inductor 2,2′-azinobis-(3-ethylbenzothiazoline-6-sulfonic acid) (ABTS) was purchased from Fluka (St. Louis, MO). Acetonitrile, formic acid and methanol were purchased from Sigma-Aldrich. Quercetin-3-*O*-glucoside, quercetin 3-*O*-galactoside, (–)epicatechin, procyanidin B2 and vitexin were purchased from Extrasynthese (Lyon, France).

### Polyphenolic Composition

Polyphenols of the extracts were identified using an ACQUITY Ultra Performance LC^TM^ system (UPLC) with binary solvent manager (Waters, Milford, MA) and a Micromass Q-Tof micro–mass spectrometer (Waters, Manchester, UK), equipped with an electrospray ionization (ESI) source that operates in negative and positive modes. For instrument control and data acquisition and processing, the MassLynx^TM^ software (version 4.1; Waters, Milford, MA) was used. Individual polyphenols were separated using a UPLC BEH C18 column (1.7 μm, 2.1 × 50 mm; Waters, Milford, MA) at 30 °C. Samples (10 μl) were injected and elution was completed in 15 min with a sequence of linear gradients and isocratic flow rates of 0.45 ml min^−1^. The mobile phase was composed of solvent A (4.5 % formic acid, v/v) and solvent B (100 % acetonitrile). The program began with isocratic elution with 99 % A (0–1 min), and then a linear gradient was used until 12 min, lowering A to 0 %; from 12.5 to 13.5 min it was returned to the initial composition (99 % A) and then held constant to reequilibrate the column. Analysis was carried out using full-scan, data-dependent MS scanning from 100 to 1,500 *m*/*z*. The mass tolerance was 0.001 daltons, and the resolution was 5.000. Leucine enkephalin was used as the internal reference compound during ESI-MS accurate mass experiments and was permanently introduced via the LockSpray channel using a Hamilton pump. The lock mass correction was ±1.000 for Mass Window. All time-of-flight MS chromatograms are displayed as base peak intensity chromatograms and scaled to 12,400 counts per second (=100 %). The effluent was led directly to an electrospray source with a source block temperature of 130 °C, a desolvation temperature of 350 °C, a capillary voltage of 2.5 kV and a cone voltage of 30 V. Nitrogen was used as the desolvation gas at a flow rate of 300 l h^−1^.

Single components were characterized via retention time and accurate molecular masses. Each compound was optimized to its estimated molecular mass [M-H] in the negative and positive modes before and after fragmentation. The data obtained from UPLC-MS were subsequently entered into the MassLynx 4.0 ChromaLynxTM Application Manager software. Based on these data, the software is able to scan different samples for the characterized substances.

### Osmotic Resistance

Experiments were performed on fresh pig blood. Full blood was centrifuged for 3 min at 2,500 rev min^−1^ at 4 °C, to remove the plasma and leukocytes. The erythrocytes obtained were washed thrice with a cooled (at 4 °C) phosphate-buffered saline isotonic solution (310 mOsm). Next, a red blood cell (at 2 % hematocrit) suspension containing hawthorn extracts of 0.01 mg ml^−1^ concentration was prepared and left for 1 h at 37 °C with continuous stirring. After this modification, the suspension of erythrocytes was centrifuged for 15 min at room temperature in order to remove the supernatant from the extract solution. From the cell sediment 100-μl samples of the extract-modified cells were taken and suspended in test tubes containing NaCl solutions of 0.5–0.86 % concentration and an isotonic NaCl solution (0.9 %). In solutions of the same concentrations were also suspended unmodified red blood cells that constituted the control for osmotic resistance determinations. Then, the suspension was stirred and centrifuged under the above-stated conditions. After that, the percentage of hemolysis was measured with a spectrophotometer at *λ* = 540 nm wavelength. On the basis of the results, the relation was determined between the percentage of hemolysis and the NaCl concentration in the solution. Next, using the obtained plots, the NaCl percent concentrations that caused 50 % hemolysis (EC_50_) were found. The EC_50_ values were taken as the measure of osmotic resistance. If a determined NaCl concentration was higher than that of control cells, the osmotic resistance of the erythrocytes was regarded as lower and vice versa.

### Packing Order

Packing density in the hydrophilic part of lipids of the erythrocyte membrane was studied with the fluorimetric method, using the Laurdan fluorescent probe. Measurements were made with a Cary Eclipse spectrophotometer (Varian, Palo Alto, CA). The study was carried out on isolated, unsealed erythrocyte membranes. The amount of erythrocyte membrane in the samples was determined on the basis of protein concentration using the method of Bradford (1976), the assay result being about 100 μg ml^−1^, while the concentration of the fluorescent probe was ~1 μM. To samples containing erythrocyte ghosts and the probe in a buffer solution of pH 7.4 were added appropriate amounts of hawthorn extract to obtain concentrations 0.01–0.1 mg ml^−1^. The Laurdan probe excitation wavelength was *λ*_*exc*_ = 360 nm, and the emission wavelengths were *λ*_*b*_ = 440 nm and *λ*_*r*_ = 490 nm.

Packing density in the hydrophilic part of lipids of the erythrocyte membrane was determined on the basis of generalized polarization (GP) of Laurdan, calculated with the formula (Parasassi et al. 1998):

1 $$ {\text{GP}}\, = \,\frac{{I_{\text{b}} - I_{\text{r}} }}{{I_{\text{b}} + I_{\text{r}} }} $$

where *I*_b_ is fluorescence intensity at *λ* = 440 nm and *I*_r_ is fluorescence intensity at *λ* = 490 nm.

Increased values of GP signify increased packing density of the membrane lipid polar heads, whereas decreased values of GP indicate decreased polar group packing arrangement of the erythrocyte membrane lipid bilayer.

### Erythrocyte Morphology

For investigation with the optical microscope, red cells separated from plasma were washed four times in saline solution and suspended in the same solution but containing 0.01 and 0.1 mg ml^−1^ of the extracts from the leaves and bark of hawthorn. Hematocrit of the erythrocytes in the modification solution was 2 %, the modification lasting 1 h at 37 °C. After modification, erythrocytes were fixed with a 0.2 % solution of glutaraldehyde. After that, red cells were observed under a biological optical microscope (Eclipse E200; Nikon, Tokyo, Japan) equipped with a digital camera. The photographs made it possible to count erythrocytes of various shapes, and then the percent share of the two basic forms (echinocytes and stomatocytes) in a population of ~800 cells was determined. The individual forms of erythrocyte cells were ascribed morphological indices according to the Bessis scale (Bernhardt and Deuticke 2003), which for stomatocytes assume negative values from −1 to −4 and for echinocytes from 1 to 4.

Before observation with a scanning electron microscope, erythrocytes were fixed for 48 h in 2.5 % glutaraldehyde. Then, the preparations were washed in a phosphate buffer for 20 min. Next, to dehydrate the samples, they were washed in an acetone solution of various concentrations (30, 50, 60, 70, 80, 90 and 100 %). Each sample was washed for 15 min in solutions of the above concentrations, the sample being left for 30 min in the highest concentration. Dehydrated samples were dried for 12 h at room temperature. Erythrocytes thus prepared were then deposited on the object stage and subjected to microanalysis with a Bruker (Billerica, MA) AXS Quantax Roentgen microanalyzer that operated with the program ESPRIT (version 1.8.2). After microanalysis, samples were coated with gold with a Scancoat 6 (Edwards, London, UK). The ultrastructure of erythrocytes was analyzed using an EVO LS15 (Zeiss, Oberkochen, Germany) scanning microscope equipped with an SE1 detector, under high vacuum at accelerating voltage (EHT = 20 kV).

### Oxidation Test

The antioxidant activity of the extracts was determined using spectrophotometric and fluorimetric methods. In the first method the oxidizing agent was UVC radiation (*λ* = 200–280 nm) and the object of study was erythrocyte membranes and natural lipids extracted from the membranes. In the fluorimetric method two oxidizing agents were used, UVC radiation and an AAPH oxidation inducer; the object of study was erythrocyte membranes.

Erythrocyte ghosts and lipids extracted from erythrocyte membranes were suspended in isotonic phosphate buffer solution of pH 7.4. Control samples contained erythrocyte ghosts or lipids only, whereas appropriate amounts of extracts were added to the remaining samples. The ghosts and lipid suspensions were oxidized with UVC radiation of 3.5 mW cm^−2^ intensity, emitted by a bactericidal lamp. The measure of lipid oxidation was the concentration of malondialdehyde (MDA) released during lipid peroxidation. Its concentration was determined spectrophotometrically (UV–Vis, Varian) owing to the color reaction between MDA and thiobarbituric acid (TBA). In this method the measure of membrane lipid peroxidation is the concentration of MDA released during oxidation. The color MDA–TBA reaction allows one to determine the concentration of MDA from its light absorption at 535 nm. The percentage of lipid oxidation in the presence of different concentrations of the extracts and for a fixed irradiation time of 60 min was calculated from equation 2:

2 $$ {\text{Oxidation inhibition }}\left( \% \right) \, = \,\frac{{(A_{0} - A)}}{{A_{0} }} \times 100\;\% $$

where *A*_0_ is the absorbance of the control sample and *A* is the absorbance of sample with extract.

In the fluorimetric oxidation experiments the DPH-PA probe was used. Erythrocyte ghosts, with the extracts added, were suspended in a phosphate buffer of pH 7.4 and UVC-irradiated or treated with the chemical oxidation inductor AAPH for 30 min. Free radicals, released in the process of membrane lipid irradiation, cause quenching of DPH-PA fluorescence, decreasing the fluorescence intensity. Relative fluorescence, i.e., the ratio of UVC probe fluorescence to the initial fluorescence of the probe, was adopted as a measure of the extent of lipid oxidation. The relative fluorescence of an erythrocyte ghost suspension that contained the DPH-PA probe, oxidized with UVC or AAPH radical, was assumed as a control, while the blank was relative fluorescence of a suspension of the same concentration but not oxidized.

A Cary Eclipse fluorescence spectrophotometer (Varian) was used to measure free radical concentrations in the samples. Excitation and emission wavelengths were *λ*_ex_ = 364 nm and *λ*_em_ = 430 nm, respectively. The measure of lipid oxidation was the relative change of fluorescence intensity, *F*/*F*_0_, where *F*_0_ is the initial fluorescence and *F*, the one measured during an oxidation procedure (Arora and Strasburg 1997). The percentage of lipid oxidation inhibition was calculated from the following equation:

3 $$ {\text{Oxidation inhibition }}\left( \% \right) \, = \frac{{(F_{\text{x}} - F_{\text{u}} )}}{{(F_{\text{k}} - F_{\text{u}} )}} \times 100\;\% $$

where *F*_X_ is the relative fluorescence of a UVC-irradiated sample, or oxidized by AAPH, for 30 min in the presence of extracts; *F*_U_ is the relative fluorescence of a control sample, oxidized by AAPH or UVC radiation, measured after 30 min; and *F*_K_ is the relative fluorescence of the blank sample, not subjected to oxidation procedures, measured after 30 min.

### Linoleic Acid Oxidation

Linoleic acid dispersion, prepared according to method of Surrey (1964), was added to a UV cuvette containing phosphate buffer (pH 7.4). The oxidation reaction at 37 °C was initiated by adding AAPH (Liegeois et al. 2000). Oxidation was carried out in the presence of different amounts of the hawthorn extracts. The rate of oxidation was monitored by recording changes in absorbance at λ = 234 nm brought about by the formation of conjugated bonds in oxidized linoleic acid. An UV–Vis Evolution 600 (Thermo Scientific, Pittsburgh, PA) spectrophotometer was used for the measurements. The antioxidant activity index equals the slope of the curve representing the inhibition time of linoleic acid oxidation versus antioxidant concentration.

### ABTS Bluegreen Radical Quenching

The assay was based on antioxidant-induced reduction of the blue-green radical of ABTS (ABTS^•+^) and converting it into a colorless product. The ABTS^•+^ cation radical solution was prepared by mixing 7 mmol l^−1^ ABTS and 2.45 mmol l^−1^ potassium persulfate solution at a ratio of 1:1 (v/v). The mixture was incubated in the dark at room temperature for 16 h. The ABTS^•+^ solution was diluted with water to reach an absorbance of ca. 0.9 at 734 nm. To 1,960 μl of the ABTS^•+^ solution 40 μl of diluted extracts were added, and the decay in absorbance at 734 nm was followed on the Evolution 600 spectrophotometer maintained at 25 °C by a Peltier thermostat. The decrease in absorption 5 min after addition of the solution was used for computation. The antioxidant activity of individual extracts was calculated in terms of Trolox equivalent antioxidant capacity (TEAC), i.e., micromoles of Trolox per milligram dry matter extract (Van den Berg et al. 1999; Re et al. 1999).

### Statistical Analyses

The results are presented as means ± standard deviation, calculated at a confidence level of *α* = 0.05 (*p* < 0.05) from five independent measurements. Two-factor analysis of variance was carried out. Differences between controls and compounds containing samples were found to be significant on the basis of the Dunnett test. Calculations were performed using StatSoft (Tulsa, OK) Statistica 9.

## Results and Discussion

### Polyphenolic Composition

**Fig. 1:**
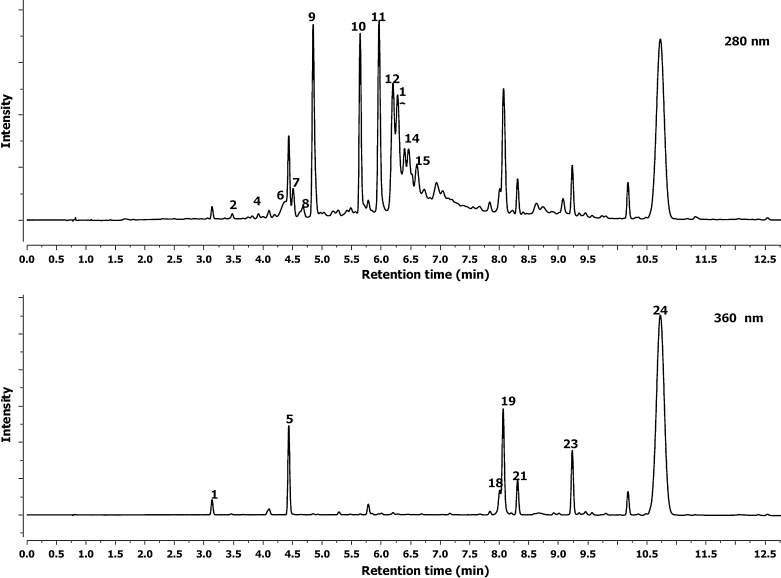
UPLC-UV 280 and 360 nm chromatograms of phenolic compounds of the hawthorn leaf preparation (refer to Table 1 for the identification of retention time peaks)

**Fig. 2:**
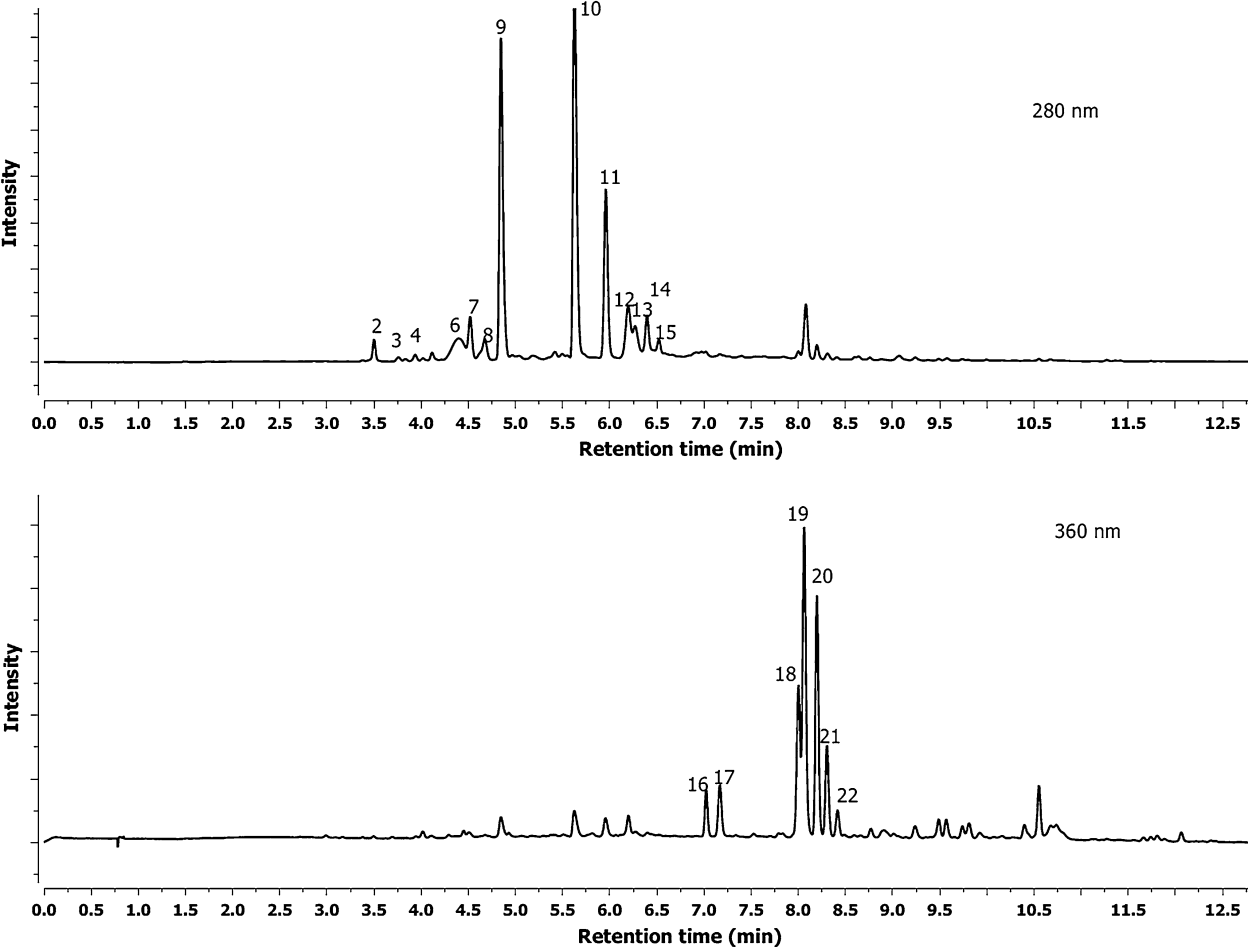
UPLC-UV 280 and 360 nm chromatograms of phenolic compounds of the hawthorn bark preparation (refer to Table 1 for the identification of retention time peaks)

The hawthorn bark and leaf preparations were analyzed by UPLC-ESI-MS-MS systems. Qualitative analysis by LC-DAD-MS-MS methods and quantitative analysis by UPLC-MS (quantified using DAD and MS detection) are summarized in Table 1, Figs. 1 and 2. A total of 20 kinds of polyphenolic compounds found in hawthorn bark and leaf preparations were identified and presented. Twelve flavan-3-ols were detected in leaf and 13 in bark preparations as (–)-epicatechin, B-type procyanidin dimers, trimers and tetramers (Figs. 1, 2). At 5.63 min retention time (Rt), compared with standard, and *λ*_max_ at 280 nm it was identified as (–)-epicatechin with the fragmentation of the negatively charged molecular ion ([M-H]-) at 289.0713 *m*/*z*. Procyanidin B2 was identified in hawthorn leaf and bark preparations. It had the same as standard procyanidins B2 (*λ*_max_ = 280 nm, ion 577.1356 *m*/*z*, fragmentation ions 289.0709 *m*/*z* and Rt 4.85 min). The other B-type procyanidin dimers, trimers and tetramers showed UV spectrum at *λ*_max_ = 280 nm, dimer ions at 577.1356 *m*/*z*, trimer ions at 865.1974 *m*/*z* and tetramer at 1,153.1768 *m*/*z*, respectively. They had the fragmentation ion at 289.0709 *m*/*z* corresponding to a monomer of catechins. The ion at 289.0709 *m*/*z* was derived from the cleavage of the link between the two-, three- or four-procyanidin monomers through fragmentation (Gu et al. 2003). Two flavonols were detected in leaf and bark preparations: quercetin-3-*O*-galactoside and quercetin-3-*O*-glucoside (Table 1, Figs. 1 and 2). Peaks 19 and 21 with Rt 8.09 and 8.35 min had *λ*_max_ values of 355 and 350 nm, respectively. Both had an [M-H] at 463.0843 *m*/*z*, and fragmentation yielded a quercetin ion at 300.0277 *m/z*. The loss of 162 amu indicates cleavage of a hexose group. This fragmentation pattern demonstrates that this peak is quercetin-3-*O*-galactoside and quercetin-3-*O*-glucoside, respectively. These compounds were described by He et al. (2008) and Hvattum and Ekeberg (2003).

**Table 1:** Percent content and characterization of phenolic compounds of the preparation of hawthorn bark and leaves using their spectral characteristic in UPLC-DAD (retention time, *λ*_max_) and negative ions in UPLC–ESI-MS

Compounds	Content bark (mg g^−1^)	Content leaves (mg g^−1^)	Rt (min)	*λ*_max_ (nm)	[MS-]	[MS-MS-]
Neochlorogenic acid	0	1.10	3.15	320	353.0866	235.9249/190.9269/146.9378
B-type PA dimer	1.54	0	3.50	280	577.1356	407.0765/289.0709
B-type PA tetramer	0.93	1.55	3.70	280	1,153.1768	865.1974/577.1334/407.0765/289.0709
B-type PA-trimer	0.77	1.35	3.90	280	865.1974	577.1334/407.0765/289.0709
Chlorogenic acid	0	11.90	4.35	320	353.0866	235.9249/190.9269/146.9378
B-type PA dimer	18.03	10.54	4.36	280	577.1356	407.0765/289.0709
B-type PA trimer	17.55	8.51	4.40	280	865.1974	577.1334/407.0765/289.0709
B-type PA tetramer	1.55	4.21	4.60	280	1,153.1768	865.1974/577.1334/407.0765/289.0709
Procyanidin B2	181.49	81.81	4.85	280	577.1356	407.0765/289.0709
(–)Epicatechin	174.82	112.32	5.63	280	289.0713	245.0814/203.0706
Procyanidin C1	89.94	54.94	5.96	280	865.1974	577.1334/407.0765/289.0709
B-type PA tetramer	44.52	55.89	6.20	280	1,153.1768	865.1974/577.1334/407.0765/289.0709
B-type PA tetramer	1.06	27.28	6.29	280	1,153.1768	865.1974/577.1334/407.0765/289.0709
B-type PA trimer	24.55	33.89	6.39	280	865.1974	577.1334/407.0765/289.0709
B-type PA tetramer	4.84	21.89	6.52	280	1,153.1768	865.1974/577.1334/407.0765/289.0709
Orientin	1.17	0	7.06	336	447.0932	357.0622/327.0512/297.0396
Isoorientin	0.92	0	7.17	336	447.0932	357.0622/327.0512/297.0396
Vitexin	2.32	45.87	8.03	340	431.0947	311.0557/283.0594/269.0435
Quercetin-3-*O*-galactoside	6.24	27.26	8.09	355	463.0843	300.0277/151.0034
Vitexin-2′′-*O*-rhamnoside	0.34	1.32	8.21	340	577.1349	431.0947311.0557/283.0594/269.0435
Quercetin-3-*O*-glucoside	1.15	15.50	8.35	350	463.0843	300.0277/151.0034
Isovitexin	2.82	0	8.42	340	431.0947	311.0557/283.0594/269.0435
Di-caffeoyl quinic acid	0	8.43	9.25	320	515.1189	353.0866/235.9249/190.9269/146.9378
Acetylvitexin 2′′-*O*-rhamnoside	0	30.31	10.75	340	619.1664	577.1349/431.0947/311.0557/283.0594
Total	576.55	555.87

Four derivatives of vitexin were detected in the hawthorn preparation. Three of them were in bark: vitexin, isovitexin and vitexin-2′′-*O*-rhamnoside. Peaks 18 and 22 (Rt 8.03 min and 8.42, *λ*_max_ 340 nm), which produced ions [M-H] at 341.0947 *m*/*z*, were identified as vitexin and isovitexin, respectively. Peak 20 with a [M-H] at 577.1349 *m*/*z* that had an Rt of 8.21 min (*λ*_max_ 340 nm) is vitexin-2′′-*O*-rhamnoside. Three derivatives of vitexin were found in the leaf preparation; two of them (vitexin and vitexin-2′′-*O*-rhamnoside) were the same as that in the bark preparation, and a new acetylvitexin-2′′-*O*-rhamnoside peak 24 (Rt 10.75 min, *λ*_max_ 340 nm) produced ions [M-H] at 619.1664 *m*/*z*. The same kind of compounds in the *Crataegus* leaves were found by Ding et al. (2010). Two compounds, 16 and 17 (Fig. 2), were detected and identified only in the hawthorn bark preparation as orientin and iso-orientin with the same ions at 447.0932 *m*/*z*. The UV absorbance, mass spectra and fragmentation mass data were compared with data obtained by March et al. (2006). Favan-3-ols ([–]epicatechin and procyanidin derivatives) were the most abundant phenolic groups found in hawthorn bark and leaf preparations; they constituted 561.59 and 414.18 mg g^−1^ of powder, respectively. The total of flavonol and flavone derivatives in the hawthorn bark extract was only 14.96 mg g^−1^ of powder, but in the leaf preparation it was much higher: 120.26 mg g^−1^ of powder. Only in leaf samples were caffeic acid derivatives found: neochlorogenic, chlorogenic and di-caffeoyl quinic acid identified by comparison with standards and UV spectrum and *m*/*z* ions which contained a total 21.43 mg g^−1^ of powder. Hawthorn bark is known to be a very good source of flavan-3-ols, derivatives of (–)epicatechin and their oligomers (Oszmiański and Bourzeix 1995).

### Osmotic Resistance

**Fig. 3:**
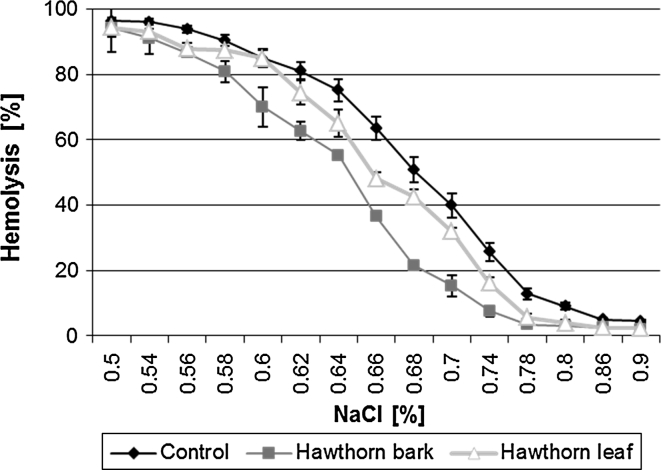
Percentage of hemolysis of cells modified with hawthorn leaf and bark extracts of 0.01 mg ml^−1^ concentration vs. sodium chloride concentration

Figure 3 shows the relation between the percentage of erythrocyte hemolysis and the concentration of sodium chloride. The research was conducted on osmotic resistance of red blood cells subjected to the action of the extracts at 0.01 mg ml^−1^ concentration for hawthorn bark and leaves. The results clearly indicate that the cell osmotic resistance increases since 50 % of the erythrocytes undergo hemolysis at hypotonic NaCl concentrations that are lower compared with control erythrocytes. This indicates that erythrocytes treated with the extracts are more resistant to osmotic pressure than control erythrocytes. As seen in the figure, in both cases the concentrations were substantially lower than those of control cells (control 0.69 %, bark 0.65 % and leaves 0.66 %).

### Packing Order

**Fig. 4:**
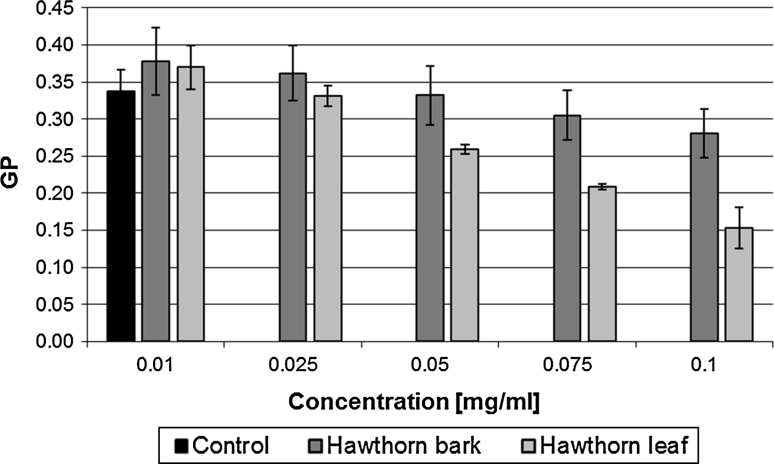
Values of generalized polarization for ghosts modified with hawthorn bark and leaf extracts

The investigation of membrane lipid packing order using the Laurdan probe was carried out on erythrocyte membranes modified with hawthorn leaf and bark extracts. For each of the extracts, experiments were conducted with five concentrations in a nonlytic range: 0.01, 0.025, 0.05, 0.075 and 0.1 mg ml^−1^, at 37 °C. The GP values were calculated using Eq. 1 and are shown in Fig. 4.

In the fluorimetric studies, a decrease in GP values with increasing concentration of the extracts was observed, which means a decrease in the membrane packing order. The substances contained in the extracts bind to the hydrophilic part of the membrane, affecting the mobility of the polar heads of lipids and thus the packing order of the hydrophilic region. It was also observed that the compounds studied alter to various extents the packing order, lower changes being induced by hawthorn bark extract and markedly greater ones by the leaf extract.

### Erythrocyte Morphology

**Fig. 5:**
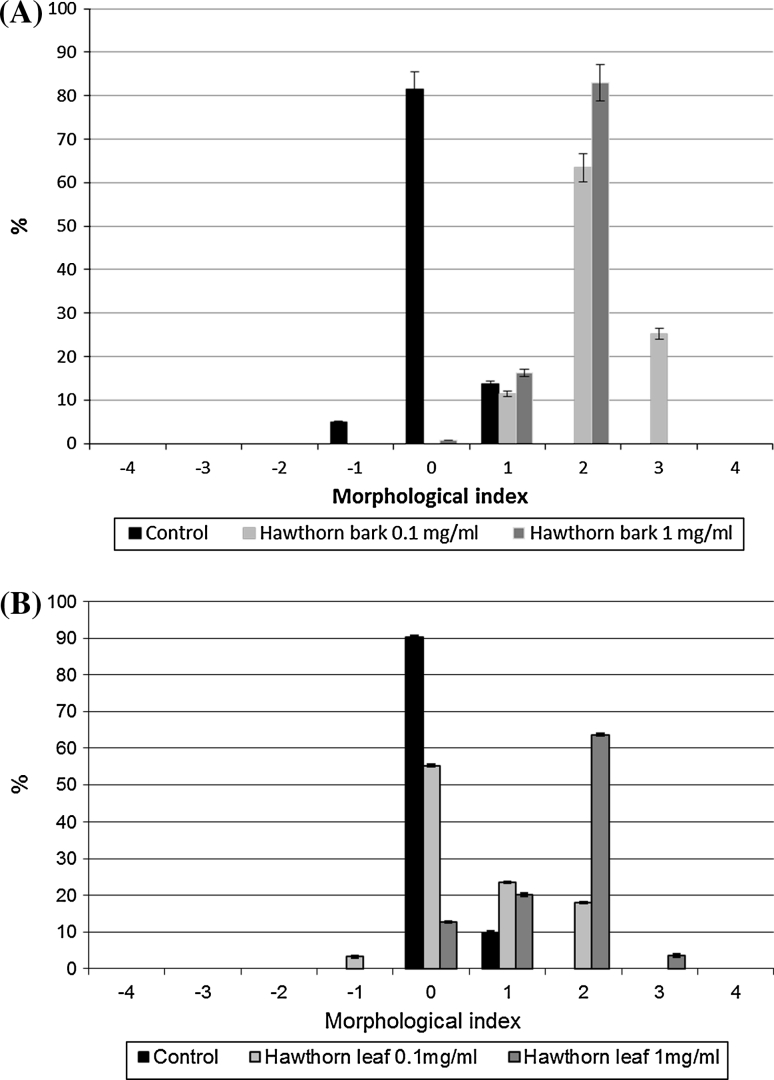
Percentage share of different forms of erythrocytes (morphological indexes) modified with different concentrations of (**a**) hawthorn bark extract and (**b**) hawthorn leaf extract

Based on microscopic observations, the presence of echinocytes was observed. The cell populations undergo shape changes as a result of incorporation of polyphenols of the hawthorn extracts into the cell membrane. Extracts were applied at two concentrations: 0.1 and 1.0 mg ml^−1^. The percent shares of hawthorn leaf and bark–modified red blood cells of specific shapes, designated by the Bessiss and Brecht (Bernhardt and Deuticke 2003) morphological indexes, are given in Fig. 5. The respective shapes have been assigned specific morphological indexes as follows: spherostomatocytes (–3), stomatocytes (–2), discostomatocytes (–1), discocytes (0), discoechinocytes (1), echinocytes (2), spheroechinocytes (3), spherocytes (4).

The results indicate that the hawthorn extracts are able to penetrate the erythrocyte membrane, causing a change in the erythrocyte shape. The location of the polyphenolic compounds within the lipid bilayer was determined. The compounds induce the formation of echinocytes mainly, which indicates that they are incorporated in the outer lipid layer of the membrane. It was also found that the number of echinocytes increases with increasing extract concentration.

**Fig. 6:**
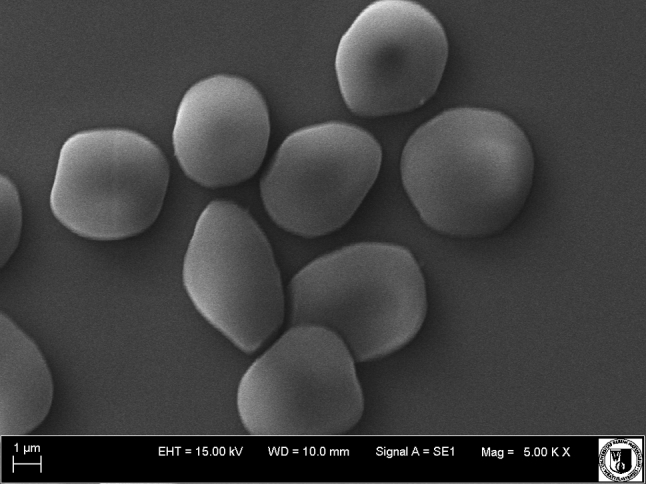
Shapes of unmodified erythrocytes

**Fig. 7:**
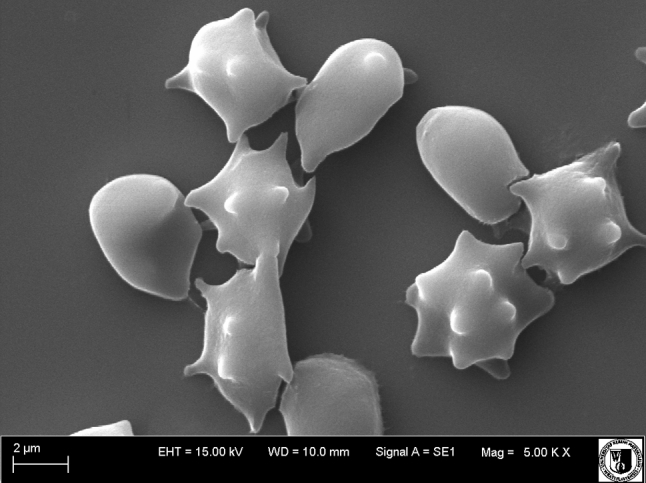
Shapes of erythrocytes modified with hawthorn bark extract

Electron microscopic images confirm the results from optical microscopy, indicating that the compounds contained in the extracts concentrate mainly in the hydrophilic part of the erythrocyte membrane, inducing the formation of various forms of echinocytes (Figs. 6, 7).

### Oxidation Test

Based on the results of oxidation of erythrocyte membranes and membrane lipids with UVC radiation, curves were drawn for the kinetics of membrane lipids and erythrocyte membrane oxidation in the presence of the substances studied for a 2-h period. The curves show a relation between absorbance, proportional to the degree of oxidation, and time of erythrocyte ghost UVC irradiation. From the figure it follows that with increasing irradiation time the absorbance, which is a measure of lipid oxidation, increases. Concentration of the extracts in the lipid suspension was 1.0–25.0 μg ml^−1^. Both the hawthorn bark and leaf extracts showed a high effectiveness in inhibiting lipid oxidation compared with the control. Based on the oxidation kinetics, for the oxidation time of 60 min, the percentage of oxidation inhibition was calculated (Bonarska-Kujawa et al. 2011; Cyboran et al. 2011). Then, a plot of inhibition percentage versus extract concentration was drawn and the IC_50_ value was read, i.e., the concentration that caused a 50 % decrease in erythrocyte membrane lipid oxidation (Table 2). Due to the difference in activities of the extracts, they were applied at different concentrations in order to obtain similar effects. From comparison of the efficacies of both the extracts it follows that hawthorn bark extract has a higher antioxidant activity.

**Table 2:** IC_50_ values for two oxidation inducers (UVC and AAPH) and two objects (erythrocyte membranes and lipids) in the presence of extracts from leaves and bark of hawthorn and for Trolox^®^

IC_50_ (μg ml^−1^)				
	Spectrophotometric studies		Fluorimetric studies	
	UVC		Erythrocyte membranes	
	Erythrocyte membranes	Natural lipids	AAPH	UVC
Hawthorn leaves	17.0 ± 5.6	34.0 ± 3.7	3.4 ± 0.7	39.0 ± 9.0
Hawthorn bark	13.0 ± 3.3	4.2 ± 0.8	2.2 ± 0.03	28.0 ± 7.8
Trolox^®^	9.0 ± 0.6	10.0 ± 1.46	3.9 ± 0.3	15.0 ± 5.0

The antioxidant activity of the extracts was also studied using the fluorimetric method. Free radicals released during membrane lipid irradiation cause quenching of DPH-PA fluorescence.

Since the studied extracts have different activities, their concentrations had to be properly selected to obtain a definite effect. When oxidation was induced with AAPH, the hawthorn leaf extract was applied at concentrations of 2.0–5.0 μg ml^−1^ and the bark extract at 1.0–3.5 μg ml^−1^. Relative fluorescence decreases with time of oxidation and concentration, the decrease being slower as the extract concentration increases (Bonarska-Kujawa et al. 2012). To compare the antioxidant activity of the substances, like in the spectrophotometric method, the IC_50_ values were determined and, together with those of Trolox, are given in Table 2. The results indicate that the bark extract is more effective at protecting membrane lipids against oxidation than the leaf extract.

In the case of membranes oxidized with UVC, the extracts when applied at concentrations 10, 25 and 50 μg ml^−1^ almost totally quenched DPH-PA probe fluorescence, which indicates total inhibition of lipid oxidation. The results obtained with spectrophotometric and fluorimetric methods indicate that the antioxidant activity of hawthorn bark extract is higher than that of leaf extract.

From Table 2 it follows that for both methods, spectrophotometric and fluorimetric, and for two oxidation inducers, UVC and AAPH, the IC_50_ value is substantially lower for hawthorn bark extract. As seen from Table 2, with the spectrophotometric method the IC_50_ concentration for hawthorn leaf extract is markedly lower for erythrocyte membranes than for natural lipids. However, the bark extract was more active toward membrane lipids. In the case of the spectrophotometric method for natural lipids and for the fluorimetric method, when AAPH is the inducer, the IC_50_ values for hawthorn bark extract are lower than those for Trolox. The low IC_50_ value indicates that the hawthorn bark extract has a high antioxidant activity compared with the leaf extract and the standard antioxidant Trolox. Different values of IC_50_ obtained with different oxidation inducers may follow from different affinity of the extracts to scavenge definite radicals that arise under UVC radiation (reactive forms of oxygen, for instance) or AAPH (organic radicals).

In the absence of a radical inducer, the rate of linoleic acid oxidation is negligible. AAPH induces oxidation, which starts at a constant rate of conjugated diene formation. Addition of an antioxidant inhibits the process for a certain time. In any case, when the inhibition time is over, the oxidation proceeds at the same rate as in the absence of an inhibitor. For each antioxidant concentration the inhibition time (T_inh_) can be determined. The measured inhibition time was directly proportional to the concentration of the antioxidant. The slope of the curve representing T_inh_ versus antioxidant concentration can be used as an antioxidant activity index (Oszmiański and Bourzeix 1995). The results show that the extract from bark inhibits linoleic acid oxidation more efficiently.

The TEAC assay was used to determine the total antioxidant capacities of various samples, usually to provide the ranking order of an antioxidant. The ABTS radical cation scavenging activity of hawthorn extracts is high. The values of Trolox equivalents are 8.65 and 12.9 μM Trolox/mg dry weight for leaves and bark extracts, respectively. Comparing the results of the TEAC assay with those of the total polyphenol content, it was found that the antioxidant activity of hawthorn extracts correlated with the total polyphenol content.

## Conclusions

The present research on the antioxidant activity of extracts from hawthorn leaves and bark toward the erythrocyte membrane, lipids isolated from the membrane of erythrocytes and linoleic acid showed high efficiency of both extracts, although a slightly higher activity was exhibited by hawthorn bark extract. This extract effectively protects lipids extracted from the erythrocyte membrane against oxidation and those contained in the membrane; thus, the whole membrane seems to be protected. These results have been confirmed both via the methods applied and for different oxidation-inducing agents. Analysis of the polyphenol composition of the extracts has shown that hawthorn bark extract contains more polyphenolic compounds than leaf extract. The high antioxidant activity of the bark extract may be due to its higher content of epicatechin, B-type PA dimer, B-type PA trimer, procyanidin B2 and procyanidin C1 than the leaf extract.

The present biophysical studies on the effect of hawthorn bark and leaf extracts on the erythrocyte membrane have shown that the polyphenolic compounds they contain become located mainly in the hydrophilic region of the membrane, inducing changes in that region. In particular, this location is confirmed by fluorimetric and microscopic studies. They showed that the extracts alter the packing order of the lipid polar heads and induce creation of echinocytes. Instead of negatively affecting the membrane structure, these changes strengthen it, making it more resistant to variations in osmotic pressure, as confirmed by the osmotic resistance investigation. It can thus be concluded that the polyphenolic compounds contained in the extracts bind to the membrane surface, constituting a barrier to free radicals which occur in the near-membrane medium. Such a mechanism can help explain the very good antioxidant properties of the extracts, which by binding free radicals deter them from penetrating the membrane and thus protect the membrane lipids against oxidation.

The studies also indicate that the polyphenol-rich plant extracts may be applied as natural, effective antioxidants that protect the biological membrane against oxidation. Moreover, the substances modulate the membrane properties in a beneficial way, decreasing the packing order of the polar heads of membrane lipids. The studies, when extended, may suggest other applications of plant extracts in medicine as they can affect membrane properties in various pathological conditions (e.g., diabetes) by, for instance, inhibiting the access of sugars to the biological membrane without any side effects.

It should be emphasized here that extracts from the leaves and fruits of this precious plant are nowadays commonly used in medicine, though not the bark extract, which has been shown here to be a much better antioxidant. That extract can also be used in many branches of industry as a preventive substance.

## Abbreviations

AAPH: 2,2′-Azobis(2-amidinopropane)hydrochloride
ABTS: 2,2′-Azinobis-(3-ethylbenzothiazoline-6-sulfonic acid)
ABTS^•+^: 2,2′-Azinobis-(3-ethylbenzothiazoline-6-sulfonic acid) bluegreen radical
DPH-PA: 3-(4-(6-Phenyl)-1,3,5-hexatrienyl) phenylpropionic acid
GP –: Generalized polarization
Laurdan: (6-Dodecanoyl-2-dimethylaminonaphthalene)
MDA: Malondialdehyde
PBS: Phosphate buffered saline
TBA: 2-Thiobarbituric acid
TCA: Tricholoracetic acid
TEAC: Trolox equivalent antioxidant capacity
Trolox^®^: 6-Hydroxy-2,5,7,8-tetramethylchroman-2-carboxylic acid
UVC: Radiation *λ* = 200–280 nm
RFT: Reactive forms of oxygen
